# Generation of wedge‐shaped dose distributions through dynamic multileaf collimator dose delivery

**DOI:** 10.1120/jacmp.v6i3.2060

**Published:** 2005-08-17

**Authors:** Jingeng Zhu

**Affiliations:** ^1^ Cancer Care Department Provena Saint Joseph Medical Center 333 North Madison Street Joliet Illinois 60435 U.S.A.

**Keywords:** dynamic wedge, dynamic multileaf collimator, intensity modulation

## Abstract

A new method of generating wedge‐shaped dose distributions through dynamic multileaf collimator dose delivery rather than computer‐controlled jaw motion is presented. The method starts with the calculation of a wedge‐shaped beam profile for the desired wedge angle. The resultant wedge beam profile is then passed to the intensity‐modulated radiation therapy (IMRT) leaf sequence generation algorithm to create multileaf collimator (MLC) segments for dose delivery. Wedge‐shaped dose distributions are achieved through dynamic MLC dose delivery guided with the generated MLC segments. The method has been tested by generating wedge‐shaped doses for a set of conventional wedge angles (i.e., 15°, 30°, 45°, and 60°). Film dosimetry is used for dose distribution verification. For each dose delivery guided with MLC segments created for the indicated wedge angle, the desired wedge‐shaped dose distributions are observed. It is concluded that the dynamic MLC can be used to implement dynamic wedges in the clinic. This technique is different from the virtual wedge or the dynamic wedge developed for a particular type of LINAC. The same method can be applied to any machine equipped with a MLC. Other advantages are that it can generate a wedge field at an arbitrary orientation as the omni wedge does, and it creates wedged and shaped fields using a MLC only.

PACS number(s): 87.53.Mr.

## I. INTRODUCTION

Generating wedge‐shaped dose distributions through computer‐controlled collimator jaw motion on a LINAC has been implemented in the clinic.^(^ ^1^ ^–^ ^4^ ^)^ Kijewski et al.^(^ ^1^ ^)^ first proposed the idea of moving the collimator jaw of the LINAC during dose irradiation to generate wedge‐shaped dose distributions. Leavitt et al.^(^ ^2^ ^)^ introduced the dynamic wedge techniques using computer control of both collimator jaw motion and dose segmentations. It was then implemented in the clinic on a Varian system.^(^ ^3^ ^,^ ^4^ ^)^ The dynamic wedge has advantages over the conventional physical wedge: (1) it eliminates the physical wedge; (2) it can generate any arbitrary wedge angle; and (3) it provides better dose distributions of straight isodose line without beam hardening.

The dynamic wedge (DW) technique generates wedge‐shaped doses by moving the collimator jaw according to the precalculated segmented treatment tables (STTs) for different beam energies, field sizes, and wedge angles. While it has been successfully implemented in the clinic, it has the disadvantage of maintaining a large set of STTs in both dose calculation and radiation delivery. The enhanced dynamic wedge (EDW) implementation reduced the number of STTs by maintaining the STTs of maximum field size only. Nevertheless, it requires real‐time calculation of STTs for the delivering field size during dose calculation and delivery. Neither DW nor EDW takes advantage of a multileaf collimator (MLC) equipped on the modern LINAC. In addition, the DW/EDW is limited to a particular type of LINAC.

The MLC technique was originally introduced to replace the cast block for beam shaping.^(^ ^5^ ^)^ Its utilization has recently been extended to deliver intensity‐modulated radiation therapy (IMRT) doses.^(^ ^6^ ^,^ ^7^ ^)^ Due to the intensive usage for IMRT, the mechanic and dosimetric features of MLC have been greatly improved. The dose calculation and quality assurance procedures have been well established. The MLC has been proven to provide high‐quality radiation doses with complex modulated beam intensity.

In this study, a wedge‐shaped dose‐generating method using a MLC is developed. The purpose of the study is to develop a technique using a MLC rather than the collimator jaw to implement dynamic wedges. The procedure includes the calculation of a wedge profile for the desired wedge angle according to the wedge definition, the creation of MLC segments based on the calculated wedge profile, and the dose delivery using the LINAC dynamic MLC technique guided by the MLC segments. This technique was tested by generating wedge‐shaped dose distributions for the conventional wedge angles (i.e., 15°, 30°, 45°, and 60°). Dose measurements using radiographic film sandwiched in a polystyrene phantom were made to obtain the dose distributions delivered with MLC segments. The study demonstrates that the approach can provide clinically desirable wedge‐shaped dose distributions for various wedge angles. The advantages of this method over the dynamic wedge are the following: (1) it is not limited to any particular type of LINAC; (2) it can generate a wedge field at an arbitrary orientation as the omni wedge does; and (3) it creates wedged and shaped fields using a MLC only.

## II. METHOD AND MATERIALS

### A. Calculate the wedge profile for the desired wedge angle

Generating wedge‐shaped dose distributions through the IMRT MLC segmentation technique requires a wedge profile for the desired wedge angle. According to the definition of the International Commission on Radiation Units and Measurements (ICRU),^(^ ^8^ ^)^ the wedge angle refers to “the angle through which an isodose curve is titled at the central ray of a beam at a specified depth.” The recommended wedge definition depth is 10 cm. Therefore, the wedge profile for the desired wedge angle has to be calculated from the isodose curves.

The calculation of wedge profile from isodose curves is a two‐step procedure. The first step is to calculate the wedge transmission factor (WTF) based on the isodose curves of the desired wedge angle and that of an open field with corresponding field size. The second step is to multiply the WTF by the profile of the open field to obtain the profile of the wedged field.

**Figure 1:**
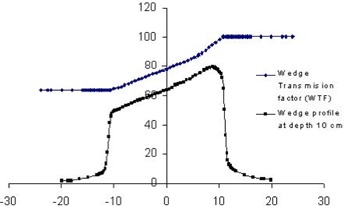
A set of wedge transmission factors (WTF) and the corresponding wedge profile calculated for a 30° wedge of a 6 MV, 20×20 cm field

The method of the physical wedge filter design described by Aron and Scapicchio^(^ ^9^ ^)^ is used to calculate the WTF. The principle of this method is to determine the ratio of the percent depth doses of wedged and open fields at various points. On an isodose chart of open field, a line is drawn at the wedge definition depth across the field at a right angle to the field central axis. A series of parallel lines is drawn making an angle with the field central axis equal to the complement of the desired wedge angle. These lines intersect the beam central axis at the intersection points of the open field isodose lines and the beam central axis. The ratio of percentage depth doses of the wedged and the open field at the intersection points of the fan lines and the reference depth line represents the WTF. The calculated WTF is then multiplied by the beam profile of the corresponding open field at the reference depth to obtain the wedge profile of the desired wedge angle. Figure 1 illustrates a set of calculated WTFs and the wedge profile for a 30° wedge of a 6 MV, 20×20 cm field.

### B. Create MLC segments

**Figure 2:**
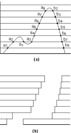
(a) A continuous intensity profile divided into relative intensity increments. The dots show the intersections of the continuous intensity profile and the parallel lines of intensity increments. The dots are grouped into ascending $(a_{i}$) and descending $(b_{i}$) dots and sorted from left to right. (b) A trajectory of a leaf pair created for delivering the continuous profile. The leaf leading and trailing positions correspond to the ascending and descending dots shown in (a).

The sliding window algorithm based on the work of Bortfeld et al.^(^ ^10^ ^)^ is used to generate MLC segments. The algorithm decomposes the wedge field dose profiles to create the unidirectional static leaf sequences for dose delivery. The intensity profile for each leaf pair is examined independently. As shown in (Fig. 2a), a continuous intensity profile is divided by a series of parallel lines representing a series of discrete intensity values. The intersections of parallel lines to the intensity profile are distinguished into two groups—ascending and descending—depending on whether the intensity profile is increasing or decreasing at the intersection. The ascending and descending intersections are sorted from left to right. The number of ascending intersections is equal to the number of descending intersections, which equals the number of MLC leaf segments to be created to produce the intensity profile. For each MLC segment, the positions of leading and trailing leaves correspond to the positions of the matching ascending and descending intersection pairs. (Figure 2b) illustrates a trajectory of a leaf pair created for the intensity profile. The overall MLC fields are created by combining the leaf positions independently generated for each leaf pair.

### C. Dose delivery technique

The dose delivery is performed by the IMRT dynamic multileaf collimator (DMLC) technique. The MLC segments created in step B are imported into the LINAC MLC controlling workstation through the network or the removable diskette. As any MLC segment file created for IMRT, the MLC segment files created for each wedged field contain the information of the number of segments, the fraction of monitor units (MUs) to be delivered through each segment, as well as the MLC leaf positions of each segment. These data are transferred to a MLC workstation to guide the dynamic MLC motions to achieve wedge‐shaped dose distributions.

### D. Verification dosimetry

Verification dosimetry was performed by sandwiching the radiographic film (Kodak XV2) in a polystyrene phantom. The film was placed in a plane that is 0.5 cm away from the beam central axis and parallel to the MLC motion to capture the resultant wedged dose distribution. The reason for putting the film 0.5 cm away from the beam central axis is to escape the MLC interleaf dose leakage. After irradiation, radiographic films were developed in the processor and scanned with a film densitometer (Model 1710, Computerized Medical System). To obtain the converting function of film density to radiation doses, a series of calibration exposures was made with the film perpendicular to the beam and at a depth of 1.5 cm in the polystyrene phantom. The doses to the calibration exposures were 5 cGy, 10 cGy, 20 cGy, 30 cGy, up to 200 cGy. Calibration films were developed in the film processor, and their densities were read and stored with corresponding doses to create the film density‐to‐dose converting function. A film dosimetry system (DynaScan, Computerized Medical System) was used to scan the film and perform the data analysis to create wedge profiles and isodose curves.

The film‐generated dose distribution reliability was verified by ionization chamber measurement on the beam central axis at depths of 1.5 cm, 5 cm, 10 cm, 15 cm, 20 cm, and 25 cm for a 10×10 cm field and wedge angles of 15°, 30°, 45°, and 60°. Each measurement delivers 100 cGy to the point at the depth of 10 cm on the beam central axis. The result indicates that the relative depth doses measured with the two techniques agree to within an average of 0.95%, with a maximum difference of 2.8% measured for the 60° wedge at a depth of 25 cm.

## III. RESULTS

The usefulness of this method to generate wedge‐shaped dose distributions through dynamic MLC dose delivery is demonstrated by creating a set of standard wedge (15°, 30°, 45°, and 60°) dose distributions for square fields of 5 cm, 10 cm, 15 cm, and 20 cm using 6 MV photons. Windows‐based software was developed to calculate the wedge profiles, to generate IMRT MLC segments for the desired wedge angles and field sizes. Procedures of dose delivery, film process, and isodose calculation were accomplished as previously described.

**Figure 3:**
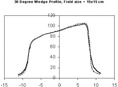
Wedge profiles for a 30° wedge, 10×10 cm filed measured at a depth of 10 cm with radiographic film. The solid line corresponds to the wedge profile resulting from the segmented MLC technique; the dashed line shows a wedge profile resulting from a conventional physical wedge.

**Figure 4:**
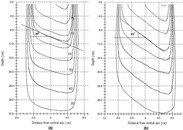
(a) Isodose distributions for a 30° 10×10 cm wedged field generated by the segmented MLC dynamic technique. Wedge angle is indicated according to the wedge definition given by ICRU report 24. (b) Isodose distributions for a 45° 15×15 cm wedged field generated by the segmented MLC dynamic technique

Figure 3 illustrates a 30° wedge profile (solid line) for a 15×15 cm field at a depth 10 cm, measured under the dynamic MLC dose delivery. A corresponding wedge profile measured with a conventional physical wedge (dashed line) for the same wedge angle and field size is displayed for comparison. Note that the wedge profiles measured at the depth of wedge definition (i.e., 10 cm) for the two methods are very close, with differences less than 2% or 2 mm in the penumbra. (Figure 4a) shows a set of dynamic MLC generated wedge isodose curves for a 30° 10×10 cm wedged field. As demonstrated in the figure, at the wedge definition depth (10 cm), our technique provides the promised wedge angle. As an additional example, (Fig. 4b) shows the isodose curves generated with the dynamic MLC technique for a 45° 15×15 cm wedged field.

## IV. DISCUSSION

This paper presents a method of generating wedge‐shaped dose distributions through the dynamic MLC technique. Comparing this method with the dynamic wedge or the virtual wedge highlights the similarities and differences. They all are implemented without using a physical wedge, which provides good beam quality and saves the therapist from having to enter the treatment room between fields. All can produce arbitrary wedge angles in the range; this offers the opportunity to improve the dose uniformity in specific clinic setups, such as the two tangential field breast cases. Contrary to the dynamic wedge or the virtual wedge developed for particular types of LINAC, this method applies generally to any machine equipped with a MLC. Another difference is that this method can generate wedged and shaped fields using a MLC only. To generate a wedged and shaped field, the IMRT leaf sequence algorithm is modified to clip the wedge profile to the blocked field aperture before conducting MLC segmentation for each leaf pair. Some other features are discussed in the following sections.

### A. Monitor unit efficiency

There are some improvements to the total number of MUs required for radiation treatment. Similar to the dynamic wedge, the wedge factor of this approach is dependent on the field size. It increases with the decrease of field size. Table 1 lists the wedge factors for a 30° wedge and various field sizes and compares them to the fixed wedge factor of the 30° physical wedge. The change in wedge factor provides the MU efficiency in wedge field dose delivery. Table 2 lists the total number of MUs required to delivery 100 cGy, 30° wedge dose to the wedge definition depth (i.e., 10 cm) for various field sizes using this approach. Note that the dynamic MLC dose delivery needs additional time to move the MLC leafs. Nevertheless, due to the continuous MLC motion with computer‐controlled dose pulsing, the total dose delivery time of this method is comparable to that of a physical wedge.

**Table 1:** Wedge factors of 30° wedge for field sizes of 5 cm, 10 cm, 15 cm, and 20 cm using the dynamic MLC compared with the wedge factor of 30° a physical wedge

Field size (cm)	5	10	15	20
Wedge factor (DMLC)	0.937	0.878	0.773	0.619
Wedge factor (physical wedge)	0.619	0.619	0.619	0.619

**Table 2:** Total monitor units required to deliver 100 cGy 30° wedge dose to field sizes of 5, 10, 15, 20 cm

Field size (cm)	5	10	15	20
Monitor units	104	111	127	158

### B. Generate a wedge field at an arbitrary orientation

This study has focused on investigating the feasibility of generating wedge‐shaped dose distributions through a dynamic MLC. Nevertheless, this method is not limited to obtaining wedged isodose profiles along the direction of MLC leaf motion. The idea of an omni wedge that produces wedge fields at an arbitrary orientation proposed by Milliken et al.^(^ ^11^ ^)^ can be implemented using a dynamic MLC. This is simply done using an algorithm by rotating the 2D wedge profile to indicate orientation before it is submitted for the IMRT MLC segmentation.

### C. MLC transmission

**Figure 5:**
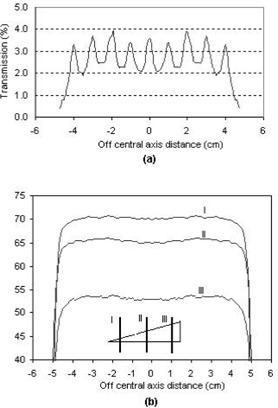
(a) Transmission through the MLC blocked field. (b) Dose profiles measured in a nonwedged direction for a 45° wedged field at three locations: I. Dose profile measured at wedge toe position of 80% field size; II. Dose profile measured at wedge middle position; and III. Dose profile measured at wedge heel position of 80% field size.

The MLC has higher dose transmission than that of the collimator jaw. This includes dose transmissions through the MLC leaf, the rounded leaf end, and the interleaf leakage. Average MLC transmission has to be measured and incorporated into the dose calculation algorithm. Rounded leaf end transmission can be overcome by closing the MLC leaves outside the treatment field. The interleaf leakage exists in the generated wedge field and cannot be taken into account in the algorithm. Nevertheless, its effect on the dose distribution diffuses in the wedged field. To illustrate this fact, a dose measurement is conducted for 15 MV X‐rays on a Varian 2100C/C LINAC (Varian Associates, Palo Alto, CA) equipped with 80 MLC leaves. For a static MLC blocked field, as shown in (Fig. 5a), the dose transmission through the MLC leaves has maximum and minimum values of 3.8% and 1.9%, and the interleaf leakage effect is 1.9%. However, for a wedged field generated by this technique, the MLC interleaf leakage effect is much smaller. (Figure 5b) shows three profiles measured in a nonwedged direction at three locations in a 45°, 10×10 cm wedged field. At wedge toe, middle, and heel positions, as curves I, II, and III present, the corresponding interleaf leakage effects are 0.2%, 0.4%, and 0.8%, respectively. This phenomenon can be explained with the mechanism of the dynamic MLC wedged dose delivery. The dynamic MLC wedged dose delivery starts from an open field and then gradually closes the MLC while the beam is on. Depending on the location, dose points along the wedged direction get different exposures. The dose point at the wedge toe location is irradiated for a longer time than staying under the MLC block, while the dose point at the wedge heel location is irradiated for a shorter time than staying under MLC block. The less time the dose point stays under MLC block, the less the interleaf leakage effect will be.

### D. Commission and quality control

The proposed approach makes use of the recent advances of the LINAC and dynamic MLC technology. This puts different demands on beam modeling, dose calculation, and quality assurance. Similar to the IMRT implementation, accurate dose calculation for the MLC‐shaped small field requires detail knowledge of head scatter, penumbra, MLC, and leaf end transmission. These factors have to be carefully measured, modeled, and commissioned into the radiation treatment‐planning and dose delivery systems, which has been furnished by most of the commercial systems due to IMRT implementation. For quality assurance, the procedure of dynamic MLC quality assurance proposed by Thomas et al.^(^ ^12^ ^)^ is sufficient for this method.

The quality control measurement of this method is the same as that for the dynamic wedge. The output factor, the central axis depth doses, and the dose profiles for selective depths have to be measured for conventional wedge angles. The recommended measurement devices are linear array detectors, film, as well as the ion chamber.

## V. CONCLUSION

This study investigated the feasibility of using the dynamic MLC technique to generate wedge‐shaped dose distributions. Experiments were conducted that successfully generated dose distributions for various standard wedge angles and field sizes. The results show that the promised wedge dose distributions were achieved for the desired wedge angles. It demonstrates the feasibility and reliability of this approach in meeting dynamic wedge requirements. This method has the same advantages as the jaw motion implemented dynamic wedge, for example, no need for a physical wedge, being able to generate in an arbitrary and continuous fashion, as well as providing better beam quality with straight isodose curves and no beam hardening. In addition, this method is not limited to any particular type of LINAC, it can generate wedge fields at arbitrary orientations as the omni wedge does, and it creates wedged and shaped field using a MLC only.
